# Natural history study of scoliosis in patients with 22q11.2 deletion syndrome, starting before disease onset

**DOI:** 10.1007/s43390-025-01193-x

**Published:** 2025-10-05

**Authors:** P. P. G. Lafranca, S. de Reuver, A. Abdi, M. L. Houben, M. C. Kruyt, K. Ito, R. M. Castelein, T. P. C. Schlösser

**Affiliations:** 1 Department of Orthopedic Surgery, University Medical Center Utrecht, P.O. Box 85500, 3508 GA Utrecht, The Netherlands; 2 Department of Pediatrics, University Medical Center Utrecht, P.O. Box 85090, 3508 AB Utrecht, The Netherlands; 3 Department of Biomedical Engineering, Eindhoven University of Technology, P.O. Box 513, 5600 MB Eindhoven, The Netherlands

**Keywords:** Scoliosis, 22q11.2 Deletion Syndrome, Natural history, Longitudinal, Progression rate

## Abstract

**Purpose:**

To date, natural history studies on scoliosis development describe only curve progression but do not include its initiation. Around 50% of children with 22q11.2 Deletion Syndrome (22q11.2DS) develop a scoliosis. Longitudinal data from a large cohort of 22q11.2DS patients is available. This study aims to inventory the natural history of scoliosis development, starting before curve onset, in 22q11.2DS patients.

**Methods:**

22q11.2DS patients are biennially radiographically screened for scoliosis from age 6 to adulthood. All available radiographs were analyzed. Outcome measures were: skeletal maturity (modified Risser classification), coronal Cobb angles, curve angle fluctuation and treatment (bracing, surgery or no treatment). An evaluation was performed of scoliosis onset, risk of progression to > 30°, curve angle fluctuation and treatment.

**Results:**

722 full-spine standing radiographs of 292 patients were included. 116 (40%) of the patients developed a curve ≥ 10°, 44% of girls and 36% of boys. Thirteen (4%) progressed to a curve > 30° and seven (2%) required surgical treatment. In patients with radiographs before age 10, 49% already had a scoliosis. 22% of the patients already had a curve ≥ 10° at first visit. More fluctuation compared to a predicted trend line was seen in future scoliosis patients.

**Conclusion:**

It appeared that many 22q11.2DS patients already have fluctuating spinal asymmetry before age 10, often without progression, and that only a subset develops a severe progressive deformity. This longitudinal dataset provides the opportunity for future risk-profiling to distinguish between stable versus progressive scoliosis for the 22q11.2DS population.

## Introduction

The 22q11.2 deletion syndrome (22q11.2DS), also known as DiGeorge syndrome, is the most prevalent microdeletion syndrome in humans [1]. This syndrome has a wide range of phenotypical expressions, including cardiovascular abnormalities, immunodeficiency, developmental disabilities and orthopedic disorders [1]. In recent years, much research has been done on the orthopedic manifestations of 22q11.2DS. One of the key findings has been that approximately half of patients with 22q11.2DS that initially have a straight spine, develop a scoliosis (defined as Cobb ≥ 10°) during growth [2–5]. Moreover, it has been shown that most of the scoliotic curves in the 22q11.2DS population resemble AIS curve morphology with comparable curve progression rates [4, 6]. However, while much research has been done concerning the epidemiology and characteristics of scoliosis in 22q11.2DS patients, no longitudinal assessment of the scoliotic curve development within this 22q11.2DS population has been performed to date.

In our national 22q11.2DS referral center, patients, after being diagnosed, are biennially screened for scoliosis with a physical exam and biplanar, standing radiographs from age six to adulthood as a part of standard clinical follow-up. Screening of 22q11.2DS patients has been performed in our center since 2013, and therefore a large longitudinal database of radiographs is available. Some of the 22q11.2DS patients with mild symptoms are only diagnosed later in life and sometime scoliosis is the first manifestation of the underlying disease. Moreover, a large part of the cohort is still young, and therefore only has one or two follow-up instances. This is also seen in our cohort, where a part of the patients has completed follow-up from a young age until skeletal maturity, while another part started screening at a later age or had few follow-up moments. To have a clear understanding of the natural history of scoliosis in 22q11.2DS, the purpose of this study is to use this cohort to inventory the natural history of later scoliosis development, whenever possible from before disease onset.

## Methods

### Study population

This is a longitudinal cohort study on genetically confirmed 22q11.2DS patients followed in a national referral center in the Netherlands. The institutional review board approved the data collection in our 22q11.2DS registry by expedited review [4]. After diagnosis of 22q11.2DS, patients aged 0 – 18 years are biennially seen by a multidisciplinary team, including screening by an orthopedic surgeon starting at the age of 6 years (earlier on indication or later if diagnosed late). This orthopedic screening includes a physical examination and upright, full-spine, conventional radiographs (anterior–posterior and lateral) including hips and pelvis. When a new scoliosis is detected, the follow-up is intensified to every 6–12 months based on age and curve magnitude. Similar thresholds of curve magnitude are used for considering brace treatment and surgical intervention in the 22q11.2DS population compared to other scoliosis patient.

All available coronal full-spine radiographs of 22q11.2DS patients were analyzed in this study. For surgically treated patients, only postoperative radiographs were excluded. No minimal amount of follow-up time was required to be included. Patients without full-spine radiographs, with low image quality radiographs and patients who could not stand during the radiograph were excluded. Data on sex, age and initiation of brace or surgical treatment were extracted from medical records.

### Radiographic assessment

Radiographs were assessed by three trained investigators (PPGL, SR, and AA) using the picture archiving and communication system, PACS (IDS7 version 23.1.10, Sectra AB, Linköping, Sweden). Skeletal maturity was evaluated through a modified Risser classification as described by Nault et al*.*, which includes the triradiate cartilage. This classification is considered more accurate than the original Risser classification and is recommended by SOSORT and Scoliosis Research Society (SRS). Risser stages range from 0- to 5 in this classification (Risser + staging) [7, 8].

### Calculations: Cobb angles

Coronal curve angles were measured on each radiograph available in chronological order. The curve angle was measured with the Cobb method (i.e. Cobb angle), the standard method used by the SRS for quantifying the scoliotic deformity [9]. Both right- and left-sided curvature angles were measured as positive values. Scoliosis was defined as solely a coronal Cobb angle ≥ 10° at last follow-up, according to SRS definition [10]. The mean Cobb angle was calculated by using the most recent radiograph. Brace treated patients were excluded in these calculations, since their curve angle at last follow-up was influenced by treatment.

### Calculations: progression risk

Both the risk of progression to a curve angle ≥ 10° and > 30° were calculated by dividing the number of patients with a Cobb angle of 10°—30° and > 30° by the total number of included patients. The 30° threshold was chosen, based on evidence that 30° is the cutoff for functional outcome and progression during adulthood [10, 11]. The risk of curve development to < 10°, 10°-30° and > 30° based on skeletal maturity and curvature size were also calculated. Moreover, for established scoliosis, it was analyzed if the curve of ≥ 10° persisted or disappeared at later follow-up.

### Statistical analysis

All descriptive analyses were performed using SPSS 27.0 for Windows (IBM, Armonk, NY, USA) and R 4.0.3 (R Foundation for Statistical Computing, Vienna, Austria). Descriptive statistics on sex, age, years of radiographic follow-up and Cobb angle at last follow-up were extracted and compared between patients with and without scoliosis development. Graphical representations were made using R 4.0.3 (R Foundation for Statistical Computing, Vienna, Austria). Because a part of our cohort was still young and not skeletally mature, a subset analysis was performed in patients with follow-up to ≥ 16 years, since most will have reached spinal maturity at this age [12].

To study the course of coronal curve angle development, it was also evaluated how much the measured Cobb angle values varied at each follow-up moment from a predicted trend line. This degree of ‘fluctuation’ was defined as the standard deviation from the predicted Cobb angle value based on a covariance pattern model for different groups (0°–10°, 10°–30°, > 30° at last follow-up). Moreover, the fluctuation between the pre-adolescence (< 10 years) datapoints were compared between patients that later did or did not develop a scoliosis. The used covariance pattern model was built in SAS v9.4 (SAS Institute Inc., Cary, NC, USA) and corrected for differences in sex, age and group means of the initial and final Cobb angles in the age range studied.

## Results

The database contained 297 eligible patients with 780 radiographs at time of data collection. Excluded were: 1 patient without preoperative radiographs, 3 patients with low image quality, 1 patient with spinal cord injury at age 3 and all postoperative radiographs. This left 292 patients with 722 sets of full-spine radiographs for inclusion. Table 1 shows the baseline data of this group. The average age at first radiograph was 9.8 years. At time of registry evaluation, 116 (40%) patients had reached Risser stage 2 or more, 199 (68%) patients had radiological follow-up available to 10 years or older and 69 (24%) patients had radiological follow-up available to 16 years or older.

**Table 1:** Baseline characteristics of the 22q11.2DS population with and without scoliosis

	Scoliosis	No scoliosis
Number of 22q11.2DS patients	116 (40%)	176 (60%)
Sex Male Female	54 (47%) 62 (53%)	97 (55%) 79 (45%)
Disappearing Scoliosis^a^ Male Female	0 – –	21 12 (57%) 9 (43%)
Cobb angle: mean ± SD^c^ Pre-operative, no brace treatment	20° ± 13° (*n* = 112)	5° ± 3° (*n* = 176)
Treatment: Brace only Surgery	4 (1%)^d^ 7 (2%)^d^	– –
Radiological follow-up: mean ± SD	3.3 ± 2.3 years	2.2 ± 2.5 years

^a^ Defined as a Cobb ≥ 10° which disappeared at later follow-up

^b^ In a selection of patients with full-spine radiographs before scoliosis onset and during regular biennial follow-up visits

^c^ The mean Cobb angle was calculated by using the most recent (preoperative) Cobb angle measurement. Brace treated patients were excluded here, since their curve angle progression was halted

^d^ In % of all 292 inclusions


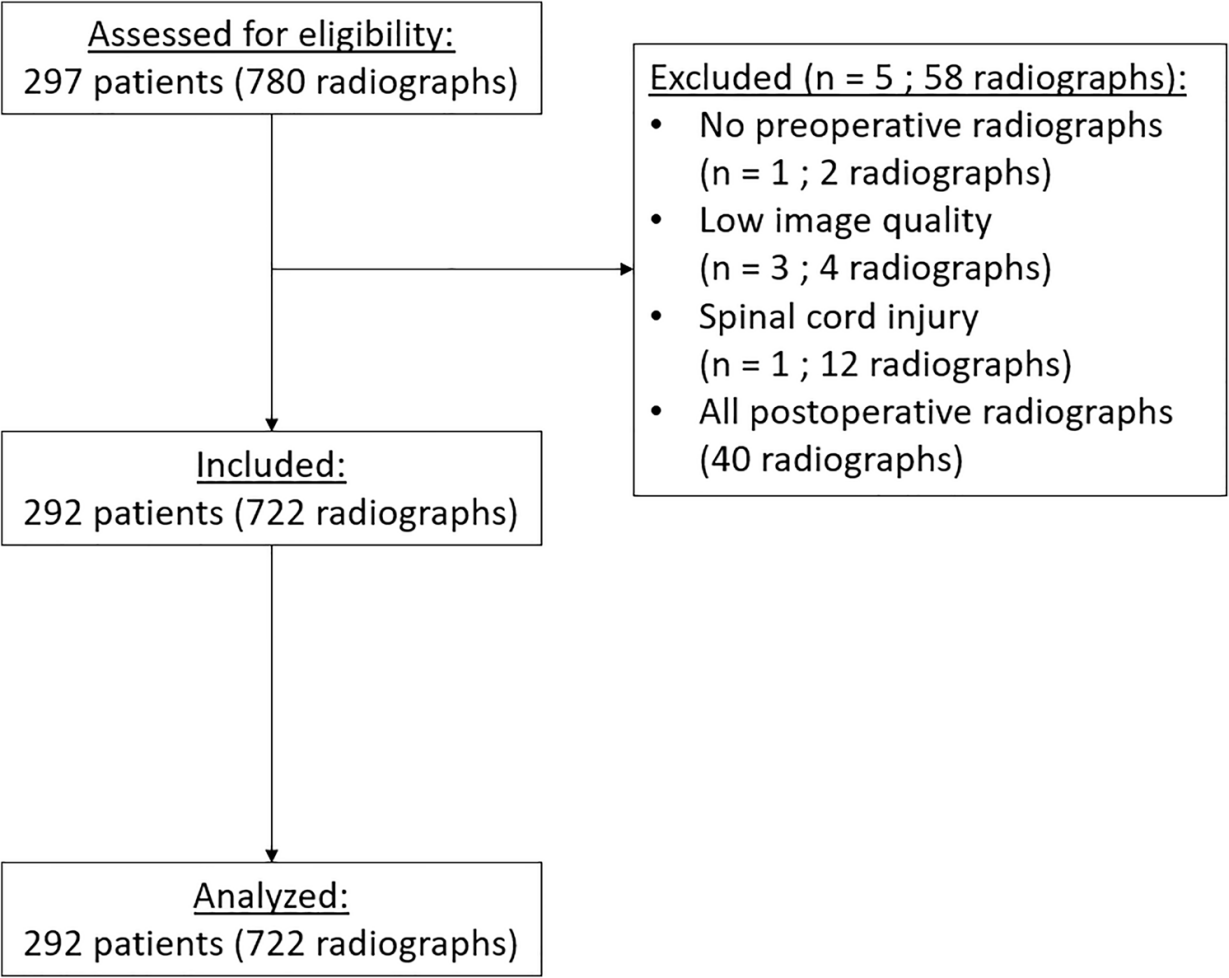


### Scoliosis development

**Fig. 1:**
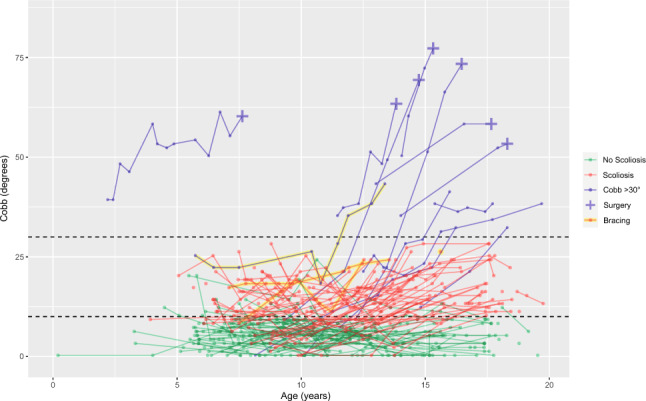
Longitudinal Cobb angle changes in 22q11.2DS patients with and without scoliosis development. Dashed lines at 10° and 30°

Coronal Cobb angle measurements of individual patients are chronologically depicted in Fig. 1. A total of 116 (40%) patients had a scoliosis ≥ 10° at last follow-up, 103 developed a curvature of 10°–30° and 13 a curve of > 30°. Fifty-four (36%) of boys and 62 (44%) of girls developed a scoliosis. Sixty-five (22%) patients already had a curve ≥ 10° at first visit. A total of 21 patients showed a scoliosis at one point, which disappeared at later follow-up. In this group with disappearing scoliosis, average Cobb angle at scoliosis diagnosis was 13° (range 24°–10°) and after disappearing 6° (range 3°–9°). Review of these patients by an orthopedic surgeon with > 30 years’ experience in scoliosis treatment confirmed Cobb angle measurements were correct and not caused by measurement error, but that most of these coronal asymmetries lacked characteristics of a true structural scoliosis and resembled a postural variation. Mean Cobb angle (± standard deviation) at last follow-up in the scoliotic group was 20° ± 13° and in the non-scoliotic group 5° ± 3°.

Of the 103 scoliotic patients that developed a scoliosis of 10°–30°, 49 (48%) were boys and 54 (52%) girls, with a mean Cobb angle of 16° ± 5°. In this group, 3 patients received brace treatment (1 boy, 2 girls). Of the 13 patients that developed a scoliosis of ≥ 30°, 5 (38%) were boys and 8 (62%) girls, with a mean Cobb angle of 53° ± 16°. In this group, seven patients had surgery (2 boys, 5 girls) and one girl received only brace treatment (Table 2). The other 5 patients were still being monitored but not treated (yet). Brace treatment is not always accepted by these patients. Tables 3 and 4 shows the prevalence of congenital heart disease (CHD) and resulting surgery. CHD prevalence is 47% in 22q11.2DS patients with scoliosis and 43% in patients without scoliosis. In both groups, CHD-related surgery is 20%. For the patients with scoliosis > 30°, 8 out of 13 cases had CHD. There were 6 cases with a curve > 60°, of which 3 had CHD and 3 had no CHD.

**Table 2:** Baseline characteristics of scoliosis in 22q11.2DS patients subdivided into scoliosis < 30**°** and ≥ 30° at last follow-up

	Scoliosis < 30°	Scoliosis ≥ 30°
Number of 22q11.2DS patients	103 (35%)^a^	13 (4%)^a^
Sex Male Female	49 (48%) 54 (52%)	5 (38%) 8 (62%)
Cobb angle: mean ± SD^b^ Pre-operative, no brace treatment	16° ± 5° (*n* = 100)	53° ± 16° (*n* = 12)
Treatment: Brace only Surgery	3 (3%) –	1 (8%) 7 (54%)
Radiological follow-up: mean ± SD	3.1 ± 2.7 years	4.8 ± 2.1 year

^a^ In % of all 292 inclusions

^b^ The mean Cobb angle was calculated by using the most recent (preoperative) Cobb angle measurement. Brace treated patients were excluded here, since their curve angle progression was halted

**Table 3:** The prevalence of CHD in 22q11.2 patients WITH scoliosis development

	Female	Male	Total
Number (*n*=)	62	54	116
CHD	31 (50%)	23 (43%)	54 (47%)
Sternotomy	10 (16%)	8 (15%)	18 (16%)
Thoracotomy	0 (0%)	1 (2%)	1 (1%)
Both Sternotomy + thoracotomy^a^	1 (2%)	3 (6%)	4 (3%)
Total surgery	11 (18%)	12 (22%)	23 (20%)

^a^ Meaning that in one patient both a sternotomy and thoracotomy were performed

**Table 4:** The prevalence of CHD in 22q11.2 patients WITHOUT scoliosis development

	Female	Male	Total
Number (*n*=)	79	97	176
CHD	35 (44%)	40 (41%)	75 (43%)
Sternotomy	15 (19%)	13 (13%)	28 (16%)
Thoracotomy	4 (5%)	3 (3%)	7 (4%)
Both Sternotomy + thoracotomy^a^	0 (0%)	1 (1%)	1 (1%)
Total surgery	19 (24%)	17 (18%)	36 (20%)

^a^ Meaning that in one patient both a sternotomy and thoracotomy were performed.

There were 175 patients with radiographs before 10 years of age. In this subgroup, 85 (49%) already had a scoliosis, therefore qualifying as Early Onset Scoliosis (EOS). A total of 69 patients had radiological follow-up available to 16 years or older. Within this subgroup 41 (59%) developed scoliosis, with a mean Cobb angle of 26° ± 18°, 11 (16%) patients had a Cobb angle > 30° and 6 (9%) needed surgery. Assessment with a Pearson Chi-Square test showed there were significantly more scoliosis cases in the group with follow-up extending up to 16 years or older compared to the group with shorter follow-up (*p* < 0.001).

### Skeletal maturity

**Fig. 2:**
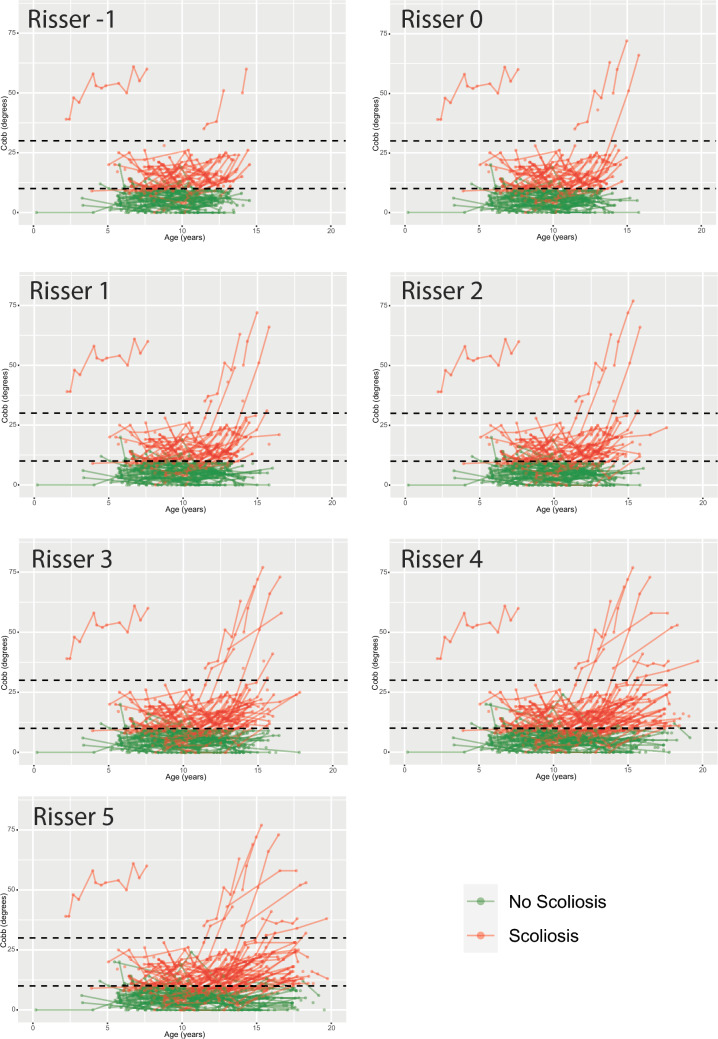
Cobb angle measurements stratified per modified Risser stage

**Fig. 3:**
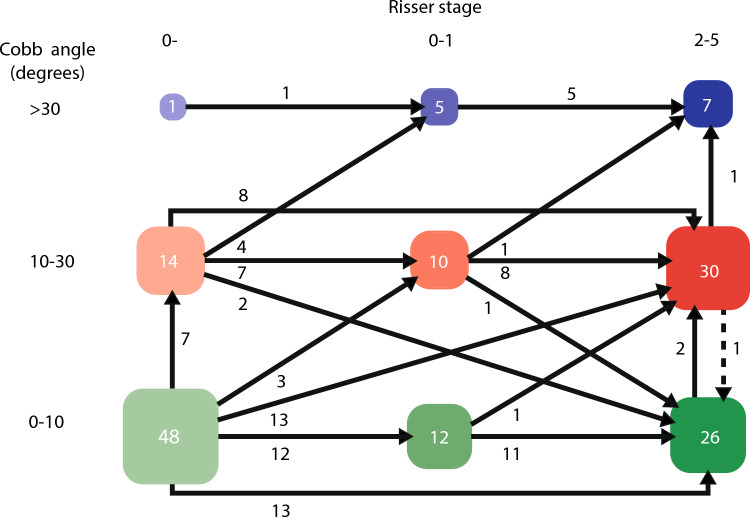
This flowchart shows the curve development of individual patients (*n* = 63) between Risser 0-, 0–1 and 2–5

In Fig. 2, Cobb angle measurements stratified per modified Risser stages are shown. It shows that most Cobb angle progression occurs at Risser 0 and 1. To visualize the curve progression rates at different stages of skeletal maturity a flowchart of the curve progression of patients who had radiographs at both Risser 0−, and 0+ or 1 and 2 or more is included (Fig. 3). Forty-eight patients had no scoliosis at Risser 0−, with 2 (4%) progressing to a Cobb angle > 30° at Risser 2–5, while of the 21 (14 + 7) curves between 10° and 30° at Risser 0−, 4 (19%) progressed to > 30°.

### Fluctuations

The covariance pattern model showed that three patient groups (0°–10°, 10°–30°, > 30° at last follow-up) showed a significant difference in Cobb angle fluctuation of to their individual groups predicted trend line, after correcting for sex, age and initial and final group means (*p* < 0.001). In this covariance pattern model, for each group a standard deviation estimate in relation to the predicted trend line was calculated, which represents Cobb angle fluctuation. Applied to data before age 10, the covariance pattern model showed significantly more fluctuation in patients that later developed a scoliosis (SD of 3.8, 5.7 and 26.1 in groups with 0°–10°, 10°–30° and > 30° at last follow-up respectively; *p* < 0.001).

## Discussion 

There are no published studies on the pathological and anatomical changes that occur during development from a normal spine into a scoliotic one. This study provides an extensive, longitudinal inventory of the natural history, from before disease onset, of scoliosis development in 22q11.2DS patients. While it is not idiopathic scoliosis, this database contains radiographs before scoliosis development, data that are currently unavailable in the AIS population. Apart from the 22q11.2DS, increased joint laxity and sagittal spinal alignment are known factors in this population that can be linked to scoliosis developmental theories [4, 13]. For the concomitant congenital heart disease (CHD) in 22q11.2DS, it has been shown that there is no relation between CHD, previous thoracotomy or sternotomy and scoliosis [3, 5, 14]. Given the fact that there is no animal model available to study biomechanical elements of scoliosis initiation and progression in fully bipedal humans, a (limited) translation of these findings to the AIS population, might also be possible [15].

Compared to earlier studies from our group, this study focusses much more on progression patterns and curve behavior within and between patients. The average age for the first radiograph was 9.8 years, which is before the start of the growth spurt, the period when most progression is taking place. In this growing population, 40% of patients developed a scoliosis, and in the patients that were followed longest, to over the age of 16 years, this was 59%.

Assessment of the fluctuation in coronal curves angles before 10 years of age revealed larger Cobb angle fluctuations in children that later developed a mild (10°–30°) or more severe (> 30°) curvature. This indicates a possible relation between fluctuation at younger age and later scoliosis development. Based on the presence and fluctuations of significant spinal asymmetry before age 10, it seems that many already have a nonstructural scoliosis at preadolescent age, which only in some individuals transforms into a significant structural and progressive scoliosis during growth. Probably for this reason, in 22q11.2DS patients with mild scoliosis (10°–30°) at skeletal immaturity, progression rate was lower (19%) than the landmark data on progression rates in skeletally immature AIS patients (60%) by Weinstein [16]. However, longitudinal data are unavailable in healthy populations. Therefore, we do not know if these nonstructural scoliotic deformities are present in the normal population and if some of them might disappear as well.

The presence of fluctuation in Cobb angle measurements (i.e. a scoliosis fluctuating in severity between different timepoints) at preadolescent age questions the ability to maintain upright spinal balance/alignment in patients with 22q11.2DS. A limitation of the standing radiographs is that several factors create subtle variations in coronal alignment, making it impossible to clearly differentiate ‘functional’ curves to mild ‘true scoliosis’. Potentially the SRS definition of a coronal curve ≥ 10 degrees on a static image at one time point is not sufficient for determining early scoliosis development. Longitudinal information on progression, or a form of flexibility assessment to quantify whether there is a structural component of the deformity, may be more helpful in future studies. In our population, the patients with less coronal asymmetry initially and less spinal asymmetry fluctuations, are less likely to develop a structural scoliosis. We are not aware of this phenomenon prior to development of the structural curves in AIS. A study by Dolphens et al*.* did show a relation between non-neutral sagittal posture and coronal trunk asymmetry in healthy subjects before the growth spurt [17]. In the 22q11.2DS population, visuomotor impairment has been reported, which also could influence the ability to regulate spinal balance, thereby possibly causing Cobb angle fluctuation [18]. Clinical AIS patients are a selection of children who already developed a progressive deformity visible by themselves, their parents or during a screening program [18]. To assess whether non-structural or postural scoliosis is also preexistent in children later developing idiopathic scoliosis, longitudinal studies using non-ionizing imaging modalities, before scoliosis development are necessary.

The SRS definition of scoliosis is a coronal Cobb angle of ≥ 10°. However, in this study there is a variety of outcomes after this 10° threshold is reached: some have rapid progressive scoliotic deformities, other mild and stable curvatures that do not need intervention and some curves even disappear. These fluctuations and the lower progression rates of the curves 10°–30°, question the validity of curve angle measurements of standing radiographs before age 10, as well as the relevance of the SRS definition for diagnosing a ‘true’ scoliosis at this age. Future natural history studies on scoliosis development may need to include dynamic assessment to evaluate curve flexibility as well as variability over time.

### Limitations

Even following from 6 years on, multiple patients showed a curve angle ≥ 10° at their first visit, meaning we are too late for pre-scoliotic imaging. Partly, this can be explained by the heterogeneity in age of diagnosis of 22q11.2DS with some patients being diagnosed with the syndrome only after their initial visit to the orthopedic clinic. Another possibility is this represents a different etiology in 22q11.2DS compared to AIS patients. Because patients were entering and leaving at various times, there was a lack of time dependent analyses. Clinical information on maturity markers apart from Risser (e.g. menses and Sanders skeletal maturity staging) were missing, and therefore we were limited in predicting the exact timing of curve progression. Detailed hematologic data next to the genetic diagnosis of 22q11.2DS was not analyzed.

In the deformities that progressed to > 30° in our cohort, most patients were over 10 years of age at their initial measurement. This limits the capability to predict later progressive scoliotic deformities with this database, and therefore predictive modelling was not performed in this study. This database does show that many patients with a Cobb ≥ 10°, also when they are under the age of 10, do not develop a severe progressive deformity. The group that best approximates skeletal maturity, i.e. with follow-up to > 16 years of age, is relatively small, and therefore our descriptive findings might change when more patients have completed follow-up to > 16 years of age. Also, only 4 patients received brace treatment and 7 surgery, and therefore the study is underpowered to draw conclusions about severe curve progression. Brace patients were excluded because their natural progression was halted, but as a result also some large curves are not included in the study.

Future steps would be to perform predictive modelling with this database and identify risk factors for later curve progression. The phenomenon of fluctuation should be further studied with repeated, supine or sitting images. Moreover, following individuals with high risk for developing idiopathic scoliosis systematically at fixed timepoints, starting before the growth spurt, could elucidate the relevance of coronal asymmetry variations prior to structural scoliosis development and the pathoanatomical changes during the earliest phase of scoliosis development.

## Conclusion

In 22q11.2DS many curves remain mild or moderate during growth. Most curve progression occurs at Risser stage 0 and 1, with greatest risk of progression to > 30° for skeletally immature patients. Before age 10, in pre-scoliotic subjects significant coronal asymmetry, fluctuating in severity, is already present.

## Data Availability

The data that support the findings of this study are available from the corresponding author upon reasonable request.
